# Torrefaction of Napier Grass and Oil Palm Petiole Waste Using Drop-Type Fixed-Bed Pyrolysis Reactor

**DOI:** 10.3390/ma15082890

**Published:** 2022-04-14

**Authors:** Syazmi Zul Arif Hakimi Saadon, Noridah Binti Osman, Moviin Damodaran, Shan En Liew

**Affiliations:** 1 Department of Chemical Engineering, Universiti Teknologi PETRONAS, Bandar Seri Iskandar 32610, Perak, Malaysia; syazmi_19001700@utp.edu.my (S.Z.A.H.S.); moviin_24137@utp.edu.my (M.D.); shan_21952@utp.edu.my (S.E.L.); 2 Higher Institution Centre of Excellence (HICoE)—Center for Biofuel and Biochemical Research, Institute of Self-Sustainable Building, Universiti Teknologi PETRONAS, Bandar Seri Iskandar 32610, Perak, Malaysia

**Keywords:** biomass valorization, Napier grass, oil palm petiole, thermochemical, torrefaction

## Abstract

The torrefaction process in the preparation of energy materials has garnered a lot of attention and has been investigated as a means of improving biomass solid fuels. The aim of this study is to study the effect of the temperature and holding time of two biomass samples: wild Napier grass and oil palm petiole. The torrefied samples are operated in a pyrolysis reactor to replicate the torrefaction procedure. The temperature parameter ranges between 220 and 300 °C while the holding time of the reaction parameter ranges from 10 to 50 min. It is found that with increasing temperature and time, the moisture content and number of O and H atoms decrease and also cause both mass and energy yield to decrease. It is found that the calorific value and the energy density increase with both parameters, which shows that optimization is needed for better solid fuel production. Between the two parameters, temperature changes have more significant effects on the torrefied samples. The optimized temperature and time are found to be 260 °C and 30 min, respectively. The usage of the pyrolysis reactor for the torrefaction reaction has been proven to serve as a good option due to similar product characteristics and equivalent results.

## 1. Introduction

Sustainable and renewable sources of energy are one of the main concerns in the upcoming years as the fear of depleting fossil fuels is growing. Among the promising sources of renewable energy is biomass utilization in the form of solid, liquid, and gas fuels. Biomass has been researched extensively for it to be partnered, mixed, and eventually become an alternative to fossil fuel. Even though it is promising, a few challenges have to be addressed for it to be competitive with current fossil fuels: (1) high consumption of energy during feedstock collection, (2) heterogenous and inconsistent composition, (3) low calorific value, and (4) difficulty in transportation [1].

Torrefaction is a thermochemical treatment in which it is carried out at a relatively low temperature of 200–300 °C and conducted in an inert environment. It is sometimes called mild pyrolysis and functions to drive out moisture and volatile matter, while at same time decomposing the polysaccharide chains. This process improves the properties of the torrefied product as compared to the original biomass, making it a better solid fuel. According to Chen et al., torrefaction possesses four main advantages: (1) increases the heating value or energy density; (2) lowers the moisture content, hydrogen-to-carbon (H/C), and oxygen-to-carbon (O/C) ratios; (3) improves the resistivity against water, and (4) enhances reactivity and grindability [2]. Torrefaction has been widely conducted using wood-based and grass-based biomass such as oil palm fruit bunches [1], willow [3], Juniper wood [4], bamboo [5], wheat [3,6], and rice husk [7] in which woody materials are more preferred as they are lignin-rich and more thermostable, but grass-based biomass has shown promise as it is more widely available and has a shorter growth duration as compared to woody materials. In most of the research in torrefaction, three main conditions have been studied to affect the performance of the torrefied material, which are biomass properties, torrefaction temperature, and duration of reaction time of torrefaction, but the latter two have more widely been analyzed. Torrefied materials can be used for the co-firing of fuel, ironmaking, and pollutant adsorbent and pretreated material for gasification and pyrolysis [8]. Despite their close thermal characteristics between torrefaction and pyrolysis, however, to the authors’ knowledge, there is no trial on utilizing the pyrolysis reactor for the torrefaction reaction process.

The biomass feedstocks of choice in this project are Napier grass and oil palm. Napier grass, which is also known as elephant grass, is a fast-growing plantation that can be found in several regions around the globe. It is classified as an herbaceous plant and belongs to the Poaceae family. As researched by Mohammed et al., Napier grass has been shown to have high volatile matter contents, heating value, and carbon content while also having low ash, nitrogen, and sulfur contents [9]. Napier grass has a lot of advantages, for example, its ability to grow in wild nature without the need of proper care, its rapid growth cycle, its high productivity, and its high ratio of energy output to energy input [10]. As Napier fulfills the criteria of an energy crop, its utilization for energy purposes should be explored further. In contrast to Napier grass, oil palm has been widely exploited for its ability to produce oil. While Napier grass production is still relatively new, oil palm has been one of the main plantations, and Malaysia has been the second largest producer of palm oil with 19.67 million tons of palm oil produced. Oil palm frond (OPF) is one of the biomass products that can be harvested from an oil palm plantation in which they are usually let to decompose for soil fertilization, erosion prevention, and nutrient recycling [11]. Although it is one of the highest contents of lignocellulosic components from the oil palm harvest, it was previously considered as waste and has been underutilized. OPF can be obtained throughout the year and accumulates to approximately 52 million tons yearly, as compared to 10 million tons and 45 million tons of produced crude palm oil and processed fresh fruit bunch, respectively, in 2018 [12]. This is concerning as OPF takes up 26.5% weight from the total oil palm source [11]. As a comparison, Napier grass from different regions around the world are produced on an average of 16 tons per hectare annually based on dry matter yield [13]. These statistics show that there is a driving factor to valorize these two sources of biomass.

The need for utilization of renewable sources is high especially toward biomass that is abundant and has higher reproducibility. Herbaceous-type biomass must also be explored for energy production to supplement and to be co-processed into wood-type biomass, which takes a longer time to reproduce. As of the time of writing, there is very little research that has been conducted on Napier grass for biofuel purposes, especially using torrefaction and pyrolysis. One of the aims for this research was to prove whether herbaceous plants such as Napier grass can be used as an energy crop in addition to the utilization of the entirety of the oil palm. This paper was also produced to highlight the ability of utilizing a pyrolysis reactor for torrefaction function with operation at lower temperatures. In this study, we focused on the torrefaction of Napier grass and oil palm petiole, which exist in abundance around Malaysia. The effects of reaction temperature and reaction time were studied with the two biomass feedstocks to observe the performance on the torrefied material. A pyrolysis reactor was used in this study in place of torrefaction to compare the results as compared to a standard torrefaction reactor.

## 2. Materials and Methods

### 2.1. Materials

Oil palm petiole (OPP) was collected from FELCRA Nasaruddin, Bota Kanan, Perak, while the Napier grass (NG) was collected from Teluk Bakong, Perak. The samples were washed, cut, and let dry under sunlight for 1 week. The raw samples were dried in an oven at 105 °C for 24 h. The dried sample was granulated to 2.5 mm in size and further grinded to about 500 μm. The lignocellulose composition of both biomass sources is shown in Table 1.

### 2.2. Torrefaction Process

**Figure 1:**
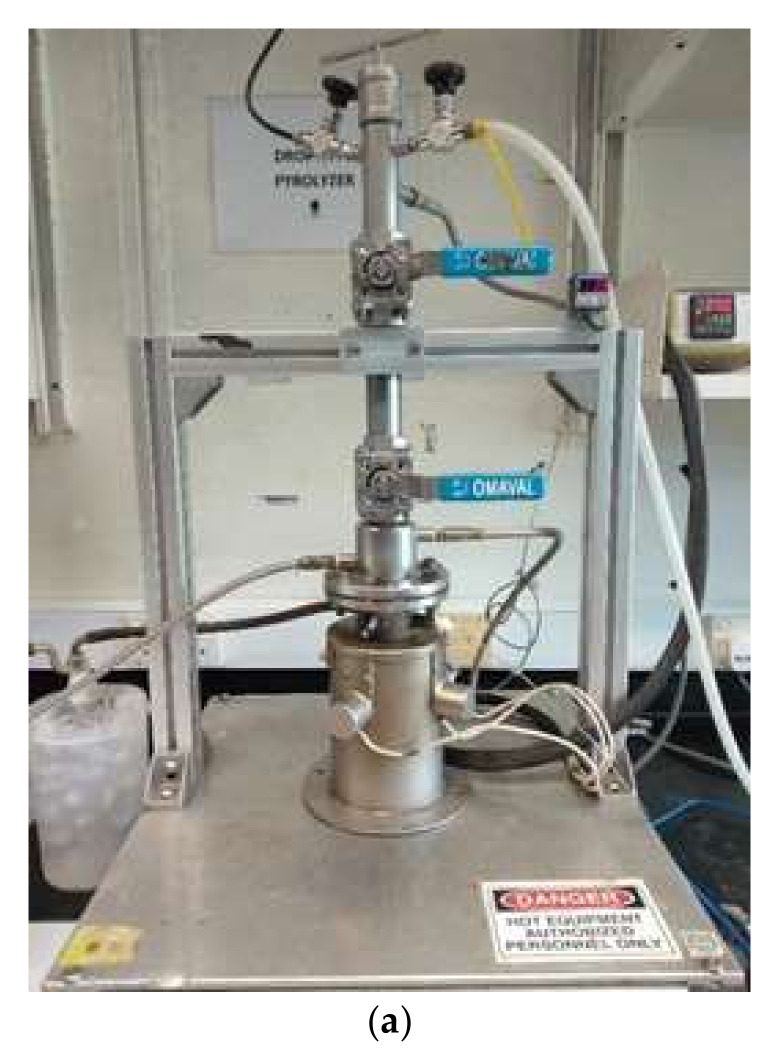

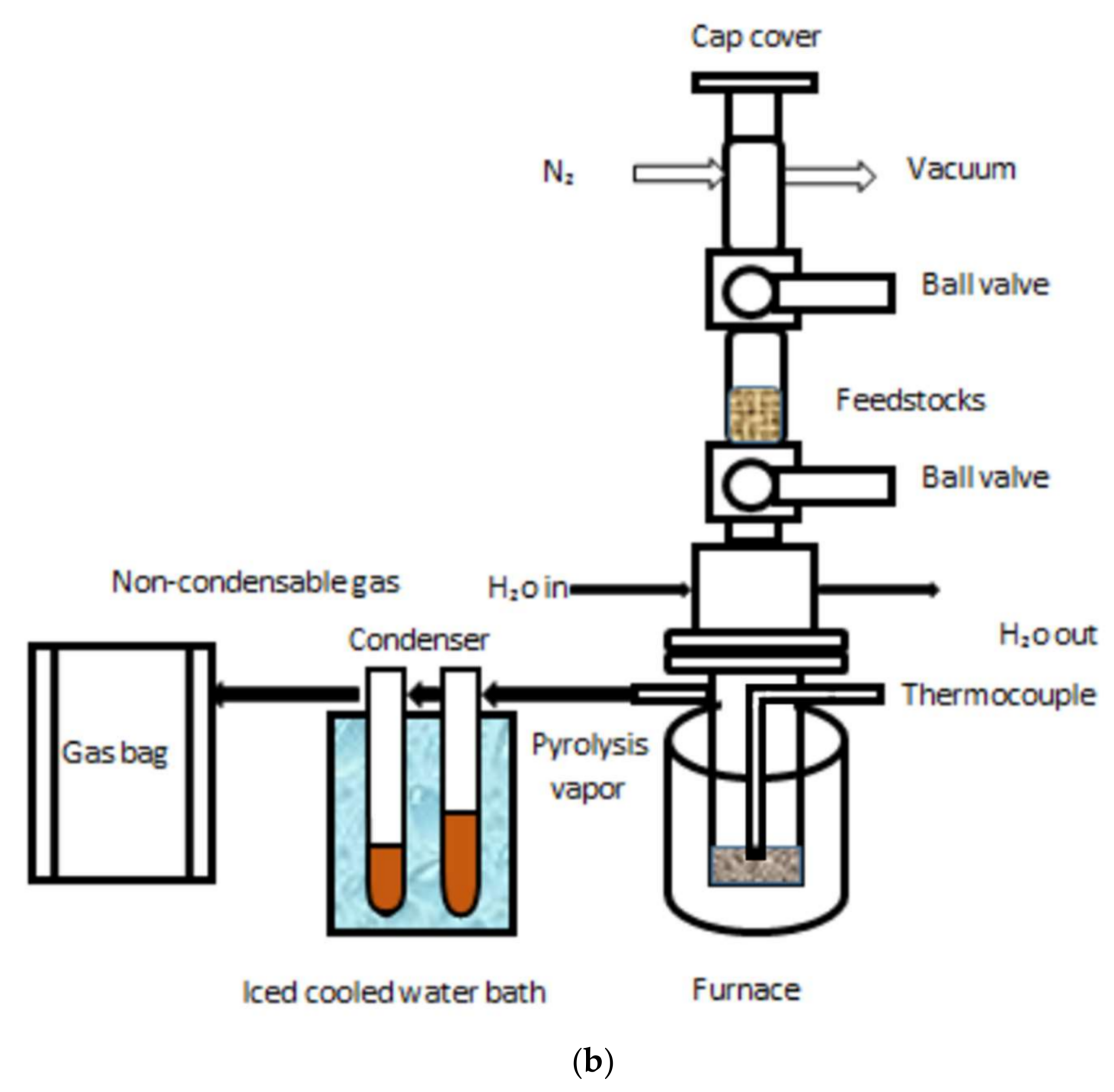
(**a**) Fixed-bed pyrolysis reactor used for the torrefaction process; (**b**) schematic diagram of torrefaction experiment in fixed-bed drop-type pyrolysis reactor.

A drop-type pyrolysis reactor was used for the torrefaction process, as shown in Figure 1. Nitrogen gas was allowed to purge the reactor for 5 min to remove oxygen from the reactor in order to prevent combustion. The reactor was calibrated before and after the sample was placed in the reactor. The reactor was heated to the desired temperature, and after the temperature was reached, the lower ball valve was let open to drop feedstock into the heating chamber. The temperature of reaction and holding time varied in the experiment, in which the latter is defined to be the duration where the temperature is held constant after the samples are dropped into the heating chamber. A total of 5 runs were conducted to both samples for 30 min of holding time with varying temperature (220 °C, 240 °C, 260 °C, 280 °C, and 300 °C). Another 5 runs were conducted with a fixed temperature of 260 °C with varying holding time (10, 20, 30, 40, and 50 min). After the holding time was achieved, the heating furnace was shut down and the samples were let to cool until room temperature before being removed from the heating chamber for further analysis.

### 2.3. Elemental Analysis

Elements of carbon (C), hydrogen (H), nitrogen (N), and sulfur (S) in the samples were determined using a LECO 932 CHNS analyzer (LECO, St. Joseph, MI, USA) in accordance with the 2mgChem80s method. It was assumed that the elements other than oxygen do not take a significant amount; therefore, the oxygen (O) content was determined by the difference of the total CHNS contents from 100%. The result from the CHNS analyzer is in the form of weight percentage; therefore, to obtain the number of atoms of the particular element, some calculations were performed using Equation (1):

(1) $$Number\;of\;atoms=\frac{w\%}{100}\times M\times \frac{1}{MW}\times AN$$

where *w%* is the weight percentage of the element, *M* is the mass of the sample, *MW* is the atomic weight of the element, and *AN* is the Avogadro’s number, which is 6.0221 × 10^23^ per mole.

### 2.4. Moisture Content and Calorific Value

The moisture content was determined in accordance with the BS EN ISO 18134-3:2016 Solid fuels-Determination of Moisture Content-Oven Dry method [14]. An amount of 1 g of sample was placed in an oven at 105 °C until a constant mass had been achieved. Constant mass is defined as the changes in mass after 1 h not exceeding 1 mg, in which up to 180 min (about 3 h) of heating for drying time is required. The sample was placed in a desiccator and later weighed. The moisture content was calculated using Equation (2):

(2) $$Moisture\;content=\frac{M_{2}\;–\;M_{3}}{M_{2}\;–\;M_{1}}\times 100\%$$

where *M*_1_ is the mass of the empty dish, *M*_2_ is the mass of the empty dish with the test sample before drying, and *M*_3_ is the mass of the empty dish plus the test sample after drying.

Calorific value (CV) is defined as the energy content, or the heating value, released during the process of complete combustion. The calorific value was determined using the BS EN ISO 18125:2017 Solid Biofuels-Determination of Calorific Value standards [15]. Approximately 0.5 g of sample was placed in a designated glass vial. A pre-calibrated cotton thread that would ignite the sample was tied-up at the ignition wire. The glass vial with the sample was brought intact to the cotton thread and the entire decomposition vessel was laced inside the bomb calorimeter. Prior to sample combustion, the weight of the sample was entered onto the screen. After 15 min of analysis, the CV results are given in terms of MJ/kg. The calorific value of the sample obtained is the higher heating value (HHV).

### 2.5. Mass Yield, Energy Yield, and Energy Density

Mass yield is defined as the percentage ratio of the torrefied sample to the raw biomass sample according to Grigiante and Antolini [16], as shown in Equation (3):

(3) $$Mass\;yield=\frac{M_{torrefied}}{M_{raw}}\times 100\%$$

where *M_torrefied_* is the mass of torrefied biomass and *M_raw_* is the mass of the raw biomass sample.

Energy yield is the usable energy in the remaining sample after the torrefaction process. It is calculated using Equation (4):

(4) $$Energy\;yield=Mass\;yield\times \frac{CV_{t}}{CV_{r}}\;$$

where *CV_t_* is the calorific value of the torrefied biomass in MJ/kg and *CV_r_* is the calorific value of the raw biomass.

Energy density is amount of energy stored in the torrefied biomass per unit mass. It is calculated as the ratio of energy yield to the mass yield, as shown in Equation (5) below:

(5) $$Energy\;density=\;\frac{Energy\;yield}{Mass\;yield}$$

## 3. Results

### 3.1. Elemental Analysis

**Figure 2:**
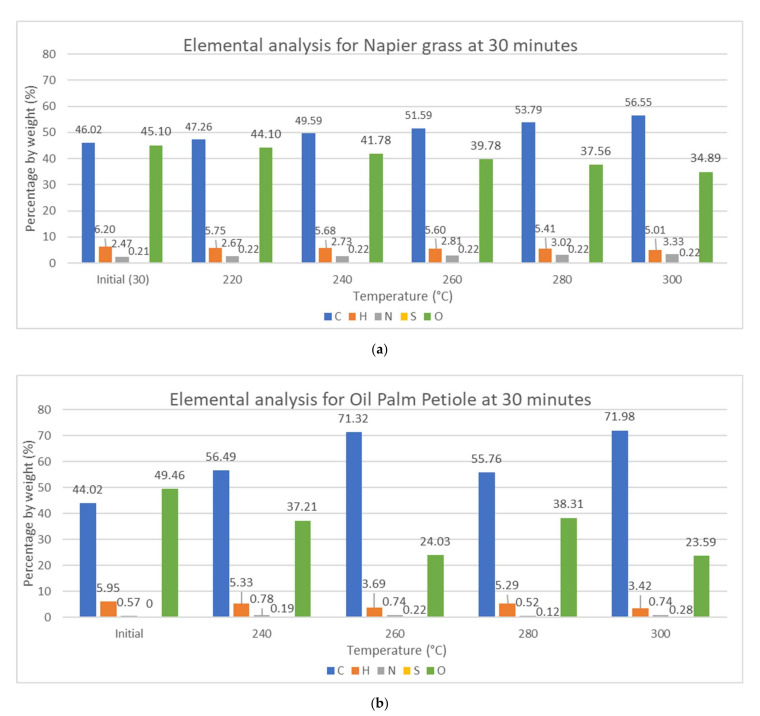
Elemental analysis at reaction time of 30 min for (**a**) Napier grass and (**b**) oil palm petiole.

**Figure 3:**
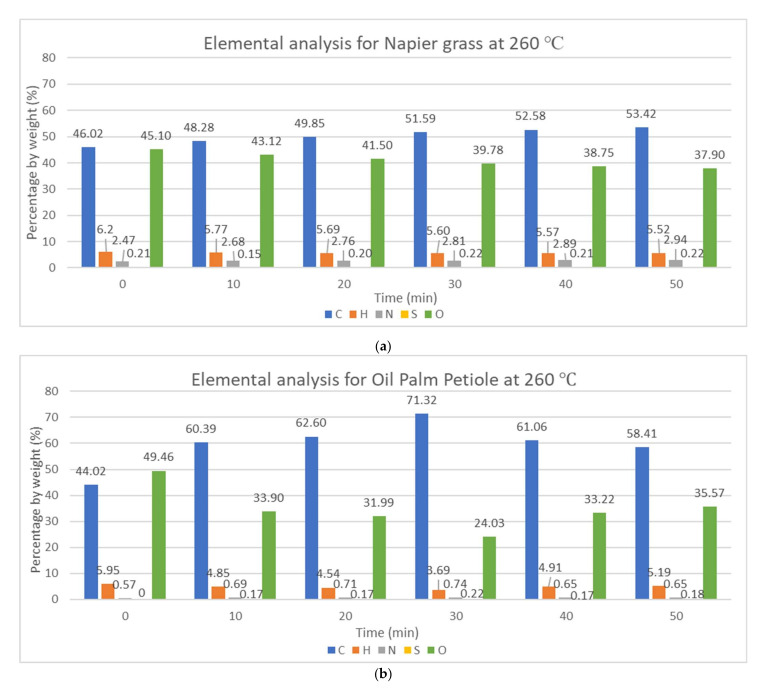
Elemental analysis at reaction temperature of 260 °C of reaction time for (**a**) Napier grass and (**b**) oil palm petiole.

Typically, there are five main elements present in the biomass composition, which are carbon (C), hydrogen (H), oxygen (O), nitrogen (N), and sulfur (S), as shown in Figure 2 and Figure 3. For Napier grass, the initial composition was taken, which consisted of 46.02 wt.% of carbon, 6.20 wt.% of hydrogen, 2.47 wt.% of nitrogen, 45.10 wt.% of oxygen, and 0.21 wt.% of sulfur, while the initial composition of OPP is reported according to literature to be 44.02 wt.% of carbon, 49.02 wt.% of oxygen, 5.95 wt.% of hydrogen, and 0.57 wt.% content of nitrogen [17]. The high composition of oxygen can contribute to fuel combustion but can also affect the calorific value. Both biomasses have low sulfur content, which means better combustion and less production of SO_x_.

### 3.2. Moisture Content and Calorific Value

**Figure 4:**
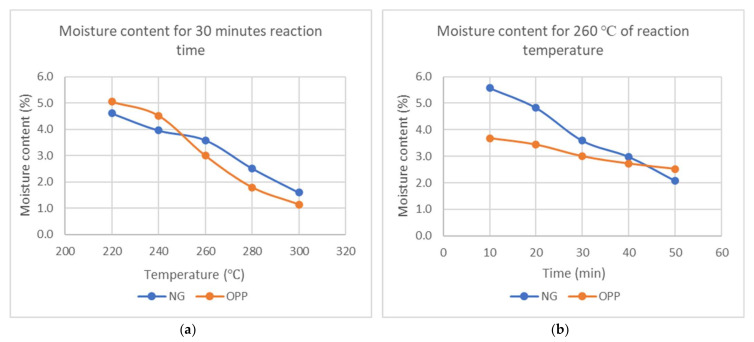
Moisture content (**a**) against temperature at constant reaction time of 30 min and (**b**) against reaction time at constant temperature of 260 °C.

It can be seen that the moisture content is inversely proportional to reaction temperature and time, as shown in Figure 4. As the temperature increases from 220 °C to 300 °C, the moisture content decreases by 66% and 78% for Napier grass and oil palm petiole from the lowest, respectively. As for the increasing reaction time from 10 min to 50 min, Napier grass loses its moisture content by 63%, while oil palm petiole loses by 31%.

**Figure 5:**
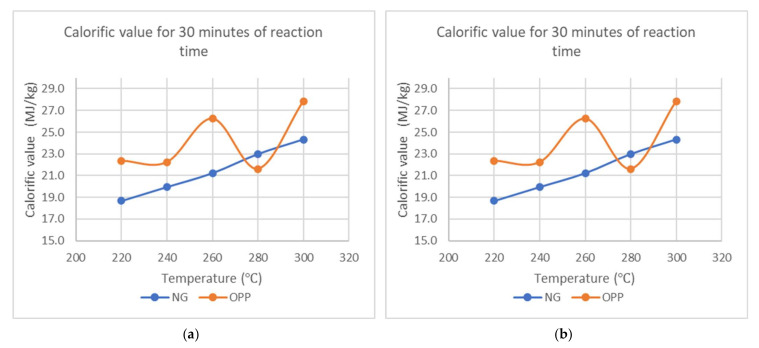
Calorific value of torrefied biomasses (**a**) against temperature at constant reaction time of 30 min and (**b**) against reaction time at constant temperature of 260 °C.

The main objective of torrefaction is to enhance the CV of biomass for it to be suitable for fossil fuel co-firing, as well as an alternative. For NG, the CV increases almost linearly with the two process variables, but the increment is not the same for OPP, where its value varies with temperature and time. The maximum CV occurs after 30 min of torrefaction in 300 °C, which is 24.33 MJ/kg and 27.84 MJ/kg for NG and OPP, respectively, as shown in Figure 5. Torrefied OPP has a higher CV, except at 280 °C where the value for OPP fell to around 21.61 MJ/kg as compared to NG having 22.98 MJ/kg.

### 3.3. Mass Yield, Energy Yield, and Energy Density

**Figure 6:**
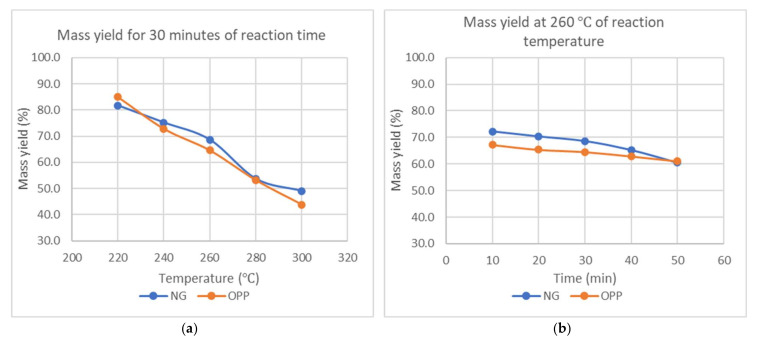
Mass yield (**a**) against temperature at constant reaction time of 30 min and (**b**) against reaction time at constant temperature of 260 °C.

For a fixed torrefaction reaction time, it can be seen that the mass and energy yield consistently decrease with temperature. The mass loss for Napier grass is slightly less than OPP by about 40% from the lowest temperature, while OPP loses its mass by 48% from the lowest temperature, as shown in Figure 6.

**Figure 7:**
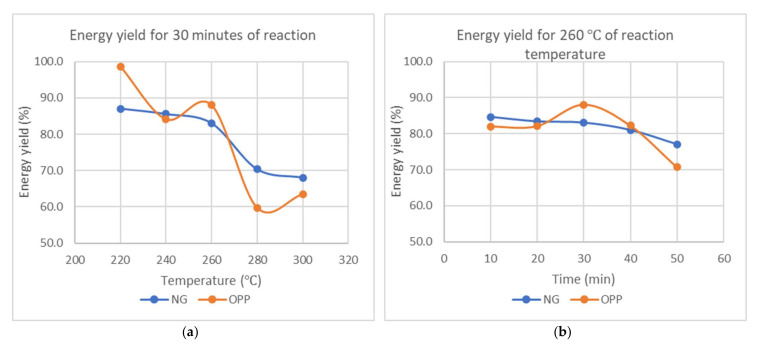
Energy yield (**a**) against temperature at constant reaction time of 30 min and (**b**) against reaction time at constant temperature of 260 °C.

Energy yield is defined as the energy remaining after the torrefaction process. From Figure 7, energy yield decreases generally when both variables increase but the reaction temperature causes a more prominent effect as compared to reaction time. The reaction time causes a significant decrease of about 21.9% as compared to 8.9% for NG and 35.6% as compared to 13.7% for OPP.

**Figure 8:**
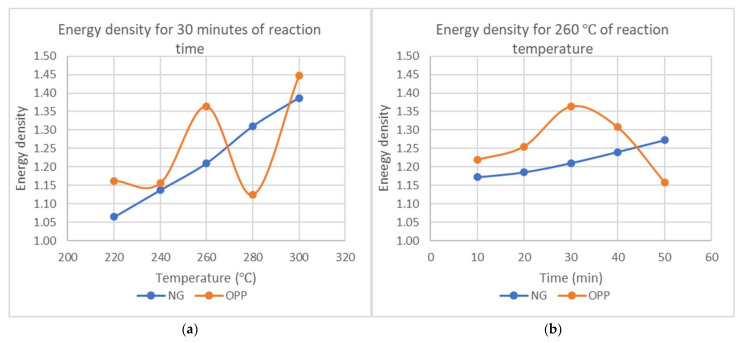
Energy density (**a**) against temperature at constant reaction time of 30 min and (**b**) against reaction time at constant temperature of 260 °C.

From Figure 8, the energy density for NG increases steadily with reaction temperature and reaction time. For OPP, as the temperature and reaction time increase, the energy density increases linearly, but after reaching 30 min, the value drops.

## 4. Discussion

### 4.1. Elemental Analysis

From Figure 2 and Figure 3, the carbon and nitrogen content increase slightly while the hydrogen and oxygen content decrease with longer reaction time and higher temperature. This is due to the breakage of C–H–O bonds in methoxy- and hydroxy form, as well as (1,4)-glycosidic bonds in cellulose, causing the liberation of water molecules and volatile matter, which also emits lipophilic extractives. Many of the carbon atoms remain in the structure upon decomposition of hemicellulose, and this increases the ash and fixed carbon content [18]. Oxygen atoms are migrated and lost in the form of H_2_O, CO_2_, and CO [19]. This result is consistent with previous studies performed by Chen et al., Uemura et al., Ivanovski et al., and Inayat et al. [1,3,20,21].

The changes in the elemental content can also be visualized using the atomic hydrogen-to-carbon (H/C) ratio and oxygen-to-carbon (O/C) ratio. Generally, the H/C ratio and O/C ratio decrease significantly with torrefaction from H/C of 0.135 for both the biomass sample and O/C of 0.980 and 1.124 for NG and OPP, respectively. The changes in H/C from the initial elemental content are in the ranges of 9.6–34.24% for NG and 26–64% for OPP, while the change in O/C ranges from 4.8 to 37% and from 39 to 71% for NG and OPP, respectively.

**Figure 9:**
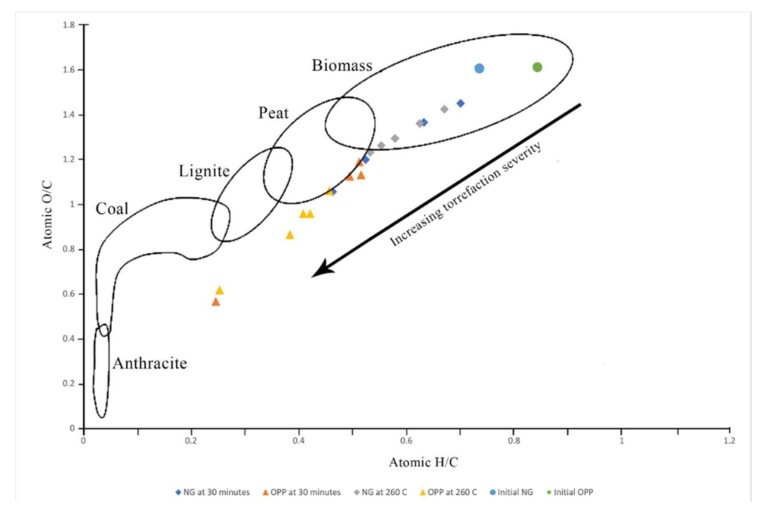
Van Krevelen diagram of the torrefied biomass.

The ratios of atomic H/C and O/C can be plotted into a van Krevelen diagram to compare the loss of hydrogen and oxygen by torrefaction. From Figure 9, the initial atomic ratios of H/C and O/C of NG and OPP are located within the biomass region, as expected. The trend shows declines in O/C and H/C when the temperature and the reaction time rise. OPP seems to have a sharper decline in H/C and O/C where the difference from the initial is quite significant and even located outside of the biomass region. For NG, torrefaction only changes the ratios slightly, in which the ratios of O/C and H/C for the torrefied NG still lie in the biomass region. It can also be seen that the effect of temperature is more notable than the effect of reaction time. This is shown by the more widely dispersed points on the van Krevelen diagram when temperature is varied. As temperature and time increase, much more hydrogen and oxygen are released as water, leaving only more carbon, which makes them closer to coal. Decarbonation occurs less severely as compared to dehydrogenation and deoxygenation, which causes decreases in H/C and O/C ratios. By torrefaction, thermal decomposition occurs toward most of hemicellulose and cellulose, while most lignin is retained within the sample, which results in lower atomic H/C and O/C ratios. This observation is similar to Granados et al. [22] and Poudel et al. [23] using poplar wood and waste wood, respectively, in which they showed a similar trend. This shows that herbaceous plant and oil palm waste can still be comparable to torrefied wood biomass. The potential of torrefied NG is relatively high as it can match other torrefied biomass feedstocks, but with its high reproducibility, more products can be produced and commercialized.

### 4.2. Moisture Content and Calorific Value

Along with the increment in temperature above 200 °C, hemicellulose decomposes, C-H-O bonds are broken, and water molecules are formed via condensation reaction. When the temperature rises above 270 °C, more devolatilization and carbonization of hemicellulose lead to more water molecules being released. This is evident in the slow reduction in moisture from 220 °C to 260 °C but rapid decline after 260 °C.

After the torrefaction process is complete, hydroxyl groups are destroyed, preventing the formation of hydrogen bonding, which makes the torrefied biomass more hydrophobic. This hydrophobicity effect is likely due to the hydroxyl group removal and formation of micropores on the surface, as reported by Chen et al. [21]. The reduction in moisture content is also attributed to tar condensation within the torrefied biomass, which also prevents moisture absorption, as reported by Felfli et al. [22]. A similar effect can also be found when other biomasses are used such as Marula seeds, blue gum wood [23], and rice husk [24].

The value for CV in OPP increases with reaction time, achieving a maximum at 30 min, and then decreases gradually. This declining trend is attributed to more decomposition of the molecular structure, which causes more decarbonization and reduction of bonding between molecules. A similar trend was also found by Baskoti et al. in which they attributed it to the release of carbon content after prolonged exposure to high temperature [24]. With the reduction in O atoms, the calorific value increases as it can inefficiently affect the combustion and energy release properties of samples. Similar values were obtained from the other torrefaction performed onto the whole oil palm frond, as well as coconut petiole [25,26]. S′wiechowski et al. also showed that the highest HHV for oxytree material can be achieved at 300 °C but suggested 200 °C to be better in terms of its economics aspect benefits [27]. From the two process conditions, the temperature seems to have the prominent effect. As OPP normally has a higher CV than NG, it shows its potential to be further processed to be a viable fuel source. Table 2 compares the results obtained to the indicative commercial solid fuels from commercial sources and biomass sources, which all show that torrefied samples are higher than typical biomass feedstock and comparable to coal.

### 4.3. Mass Yield, Energy Yield, and Energy Density

The mass loss for all samples can be attributed to the degradation and decomposition of hemicellulose, which normally occur starting from 255 °C [30]. At temperatures higher than 255 °C, most of the components that still exist would be lignin as they are the most thermally stable component. According to Chen and Kuo [31], within the torrefaction temperature ranges, hemicellulose and the residue of cellulose are the major degraded components, while lignin is only be affected slightly. This process causes decarbonization, dehydrogenation, and deoxygenation, which results in a loss of mass after the process. Within this temperature range, hemicellulose will undergo devolatilization and carbonization processes. This results in a tremendous decrease in solid mass yield. Cheng et al. [32] also proved there is an increment in torrefaction rate with temperature using wheat straw as the feedstock. Similarly, the prolonged reaction time causes a greater amount of hemicellulose to be degraded, allowing more devolatilization of components and therefore producing a larger mass loss.

Energy yields decrease generally for all samples due to the decomposition of hemicellulose, as well as condensation reaction, at these temperatures. During the process, oxygen and hydrogen are released in the form of H_2_O molecules, and the energy content is preserved in the lipids and organic compounds. As compared to other herbaceous biomass, NG shows much superior properties with a higher calorific value and greater energy yield [33].

As written in the literature, the energy density is supposed to increase with temperature [2]. This is due to the moisture and volatile matter being released, but with the fixed carbon and ash remaining in the torrefied biomass. The increment in the energy density may be attributed to the more significant decrease in mass yield. Based on the results of the yields and energy density, there is a need for optimization of parameters on torrefaction to obtain the optimal condition. Although a higher energy density is desired, the amount of usable end product must also be maximized for it to be industrially feasible. It is found that the optimal time would be at 30 min where the energy density is relatively high while also obtaining a more significant amount of product mass. This result is comparable with the result from Mamvura et al. [34]. The optimized temperature would be 260 °C in which the mass yield is substantial while also having a high energy density. This is also supported by Batidzirai et al., who mentioned that mild torrefaction is more preferable for optimal conditions according to its higher heating value, cost-effectiveness on energy usage, and adequate improvement on biomass characteristics [35].

### 4.4. Using Pyrolysis Reactor for Torrefaction

As mentioned by Chen et al. [8], torrefaction is considered as mild pyrolysis but using a lower range of temperature and with the purpose of upgrading biomass into coal-like material. As these two technologies share comparable properties, the same equipment can be used for both processes. Similar results can also be obtained from Mamvura et al. [34], where the usage of a tube furnace was employed to perform the torrefaction process onto marula seeds and blue gum wood, resulting in 275 °C and 20 min. Almost equivalent results were found by Bridgeman et al. using reed canary grass, wheat straw, and willow using a torrefaction reactor.

Torrefaction is considered to be one of the pretreatment methods for production of bio-oil from pyrolysis reaction. By using a pyrolysis reactor for torrefaction, there is no need for additional steps between the two processes. By doing so, it eliminates the need for re-purging the reaction chamber with gases. This can possibly lead to energy, time, and cost savings when the same reactor is used for both processes. Winjobi et al. proved that employing torrefaction before a fast pyrolysis does not add up to the bio-oil production cost when the net present value is zero [36], which supported the reason for using the same reactor for both processes.

The only drawback of using a pyrolysis reactor as a torrefaction reactor is the need for modification to accommodate the temperature and heating rate in accordance with torrefaction properties. As torrefaction occurs at a temperature lower than pyrolysis, the reactor must be fitted to maintain the temperature range of a torrefaction process. Despite this, the modification to the pyrolysis reactor is minor to be upgraded as compared to the purchasing cost of a torrefaction reactor. The scale-up cost for industrial use is speculated to be low as only temperature and heating control will be upgraded.

## 5. Conclusions

The feasibility of torrefaction to upgrade biomass properties as a feedstock has been evaluated. In this study, torrefaction of Napier grass and oil palm petiole with various reaction temperatures and times is exhibited. The elemental analysis has shown that there is a reduction in hydrogen and oxygen compared with the other feedstocks from van Krevelen while preserving the carbon content and increasing the temperature and time. With increasing temperature and time, moisture content was significantly reduced, making the torrefied biomass more hydrophobic. The calorific values increase linearly for NG, whereas for OPP, the value varies with temperature. The calorific value is comparable to other solid fuels and can be much higher than other biomass sources, which shows that it has an enormous potential to become a reliable solid fuel.

Mass yield and energy yield after torrefaction also decrease steadily, but a more prominent effect is seen with varying temperature. The energy density of the torrefied NG increases linearly under both conditions. For OPP, the energy density generally increases with a slight drop, while with increasing time, the energy density increases to a maximum at 260 °C followed by a gradual decline. Higher temperatures seem to produce better physicochemical properties but also return low yields; therefore, optimization is needed to maximize the advantages. There is a need for optimization using both temperature and time, in which the resulting optimized temperature and holding time are 260 °C and 30 min, respectively, due to its relatively high calorific value, lower energy usage, and adequate improvement on biomass characteristics from torrefaction pretreatment. Economic feasibility is an important aspect in evaluating a viable source of fuel in which all the parameters need to be optimized. The optimized parameters are similar to other values from the literature, which shows that the values obtained can be considered to be the best point of torrefaction.

Napier grass has been shown to be a viable candidate for biofuel feedstock as it shows promising properties comparable to other biomass sources. The usage of a pyrolysis reactor for torrefaction is found to be very feasible for undergoing torrefaction as the retrieved products exhibit properties of expected and predicted torrefied samples. As there is no modification to the reactor, this shows that torrefaction pre-treatment can be performed with any pyrolysis reactor where repurposing the reactor from pyrolysis can be more economical.

## Figures and Tables

**Table 1:** Composition of Napier grass and oil palm petiole.

Biomass Sources	Percentage %		
	Cellulose	Hemicellulose	Lignin
Napier grass	39–68	16–34	17–27
Oil palm petiole	35	18	22–25

**Table 2:** Comparison of properties of our results with indicative fuels [28,29].

Solid Fuel	Torrefied NG ^1^	Torrefied OPP ^1^	Charcoal	Coal	Wood Pellets	Saw Dust	Rice Husk	Bamboo Leaves	Coconut Husk
Moisture content (%)	1.59–5.57	1.13–5.05	1–5	10–15	7–10	13.8	7.2	7.7	13.4
Calorific value (MJ/kg)	18.7–24.3	21.6–26.2	30–32	23–28	15–16	16.9	15.6	15.7	15.9

^1^ This study.
